# Histological analysis of low dose NMU effects in the rat mammary gland

**DOI:** 10.1186/1471-2407-9-267

**Published:** 2009-08-03

**Authors:** Tessa J Murray, Angelo A Ucci, Maricel V Maffini, Carlos Sonnenschein, Ana M Soto

**Affiliations:** 1 Department of Anatomy and Cellular Biology, Tufts University School of Medicine, 136 Harrison Avenue, Boston MA 02111, USA; 2 Department of Pathology, Tufts Medical Center, 750 Washington Street, Boston MA 02111, USA

## Abstract

**Background:**

Our objective was to assess the histological changes in mammary glands of the female Wistar-Furth rat as a result of low dose exposure to N-nitrosomethylurea (NMU).

**Methods:**

Groups of 30–40 virgin female rats of between 49–58 days old received a single injection of 10, 20, 30 or 50 mg NMU/kg body weight (BW). A group of 10 control rats received 0.9% NaCl solution only. The formation of palpable mammary gland tumors was assessed weekly and, upon sacrifice at 12, 22 and 25–30 weeks after treatment, we performed a comprehensive histological analysis of all mammary gland lesions and tumors.

**Results:**

Alongside the predicted increase in tumor number and decrease in tumor latency with increasing NMU dose, we observed a number of microscopic lesions and other epithelial abnormalities in the mammary glands for all NMU doses. Two types of non-neoplastic histological changes were observed in rats exposed to 10 or 20 mg NMU/kg BW: namely, (i) an increase in the number of acinar structures often accompanied by secretion into the lumen which is normally associated with pregnancy and lactation, and (ii) an increase in the number of epithelial cells sloughed into the lumen of the epithelial ducts.

**Conclusion:**

This study establishes a baseline for low-dose exposure and defines the histological features in the mammary gland resulting from NMU exposure. Furthermore, this system provides an ideal platform for evaluating the relative susceptibility of animals protected from, or predisposed to, developing cancer through environmental influences.

## Background

The induction of mammary tumors in female rats of susceptible strains by N-nitrosomethylurea (NMU) is an established model which has been used for several decades [1]. It is the simplest method for generating a nearly complete surrogate model of human mammary carcinomas that closely mimics the human disease in terms of tumor histology and hormone dependence [1]. NMU is a direct acting carcinogen that unlike other carcinogens such as 7,12-dimethylbenz [*a*]anthracene (DMBA) [2,3] does not require the metabolic activation steps in order to form DNA adducts and has a very short half-life [4]. In addition, NMU exposure results in point mutations in codon 12 of the Ha-ras-1 gene [5]. A single NMU injection will produce 100% incidence of mammary tumors in susceptible rats and its specificity for the mammary and salivary glands is unexplained. Accordingly, numerous studies have been conducted using NMU to generate mammary gland tumors in several rat strains [6-11] including Wistar-Furth [11]. Most of these studies have used a standard dose of 50 mg NMU/kg body weight (BW) administered between 50–60 days of age. This dose is relevant for applications in which a rapid induction of tumors with a high yield is desired.

Additionally, NMU has been used to test whether animals are predisposed to neoplasia and/or susceptible to mutagens [12,13]. Perinatal exposure to estrogenic compounds induces intraductal hyperplasias in the mammary gland [14] that do not often become palpable tumors but might be induced to follow this fate by a low dose NMU challenge [12]. When using NMU as a challenge to uncover the carcinogenic effects of fetal exposure to estrogens, it is necessary to discriminate between the contributions of each agent to the histological lesions observed. "Dose-response" experiments using NMU have been performed in several different rat strains [7-9,11] but did not report histological analyses of the NMU-exposed mammary glands. In addition, when considering the differences in strain susceptibility to chemical carcinogens, a new dose response curve should be run in the strain of interest. Herein, we conducted an NMU dose-response experiment with Wistar-Furth strain rats in which they were exposed to 10, 20, 30 or 50 mg NMU/kg BW. We used 50 mg NMU/kg BW as the highest dose because previous work has shown this dose as causing the highest incidence of mammary gland tumors with the lowest level of adverse effects [8,15], and also because most work in this experimental model has been conducted with this dose. We performed a comprehensive histological analysis of all mammary gland lesions and tumors found at each NMU dose in an attempt to identify what structural changes occur in the mammary gland before and after palpable tumors become evident.

## Methods

### Animals

Virgin female Wistar-Furth rats obtained from Harlan Sprague Dawley, Inc., (Indianapolis, IN) were maintained in temperature- and light-controlled (14 h light, 10 h dark cycle) conditions in the Tufts University School of Medicine Division of Laboratory Animal Medicine. All experimental procedures were approved by the Tufts University-New England Medical Center Animal Research Committee in accordance with the Guide for Care and Use of Laboratory Animals.

### Tumor Induction and Detection

NMU was purchased from Sigma Aldrich Chemical Company (St Louis, MO) and was dissolved in warm 0.9% NaCl acidified to pH 5.0 with acetic acid (vehicle). Rats between 49 and 58 days of age received a single intraperitoneal (i.p.) dose of 10, 20, 30 or 50 mg NMU/kg BW. Solutions were used within 15 minutes of preparation. Control rats received a single i.p. injection of vehicle. In all cases an injection volume of 0.2 ml was used.

Palpation of the thoracic and abdominal-inguinal mammary glands was performed weekly starting at four weeks post-NMU injection (p.i.) to determine the incidence and latency of tumor formation. When tumors reached a size of approximately 1 cm^3 ^before the pre-determined time point for tissue harvesting, they were surgically removed and the rats were returned to the experiment. Tumor incidence was calculated as the number of animals with at least one palpable tumor or microscopic tumor lesion compared to the total number of animals in the group. Latency was defined as the time between the NMU injection and the detection of the first palpable tumor.

### Tissue harvest

Rat mammary glands were collected at 12, 22 and 25–30 weeks p.i. This was achieved by allocating the animals from all doses into 2 tissue harvest groups. In group 1 (n = 4–6), the 4^th ^and 5^th ^right abdominal-inguinal mammary glands were surgically removed under anesthesia 12 weeks p.i. At 22 weeks p.i. these animals were sacrificed and the contralateral glands were collected. Previous dose-response studies [8,9,11] indicated that 12 weeks p.i. should correlate with few palpable tumors in the lower NMU doses, while 22 weeks p.i. was selected as a midpoint between 12 weeks p.i. and 30 weeks p.i., our intended end point for the experiment.

Animals allocated to group 2 (n = 30–40) were sacrificed at 25 weeks p.i. (50 mg NMU/kg BW) or 30 weeks p.i. (all other doses) and both the 4^th ^and 5^th ^abdominal-inguinal mammary glands were collected. The animals treated with 50 mg NMU/kg BW were sacrificed earlier than the other groups as palpable tumor incidence in this group reached 100% by 17 weeks p.i. Thus, rather than subjecting the animals to surgical removal of these fast-growing large tumors and possible post-operative complications, the animals were euthanized.

### Whole mounts and histology

Tumors larger than 0.5 cm in diameter were removed from the mammary gland prior to preparing the whole mount. Tumors were fixed overnight in 10% phosphate buffered formalin and processed for paraffin embedding as described previously [15]. Sections (5 μm) were stained with Hematoxylin and Eosin (H&E).

The whole mounts were prepared following protocols described previously [16]. In brief, the mammary glands were removed and spread on a 75 × 50 × 1 mm glass slide (Fisher Scientific, Pittsburgh, PA). They were fixed overnight in 10% phosphate buffered formalin, dehydrated in alcohol, cleared of fat with toluene, rehydrated and stained with Carmine Alum. After staining, the whole mounts were dehydrated as described above, cleared in xylene, and bagged in Kpak^® ^SealPak heat-seal pouches (Kpak Corp., Minneapolis, MN) with methyl salicylate. Microscopic lesions were excised from the whole mounts and embedded in paraffin for histological evaluation. Sections were stained with H&E to visualize the overall morphology and with Trichrome stain (Masson) to assess the collagen distribution in the stroma. In addition, based on their overall morphology certain lesions were selected to undergo immunohistochemical staining using an antibody raised against α-lactalbumin (ABR Affinity Bioreagents, Golden, CO) to determine whether this milk protein was expressed in these samples. In other sections, immunostaining using an antibody raised against pan-keratin (Sigma Aldrich) was used to identify the epithelial cells within the mammary gland.

Histological sections were visualized with an Axioskop 2 Plus microscope (Carl Zeiss, Hallbergmoos, Germany) whereas whole mounts were analyzed with a Stemi-2000C stereomicroscope (Carl Zeiss). Images were captured with an AxioCam HR color digital camera (Carl Zeiss) and the Axiovision software (version 4.3).

### Statistical analysis

All statistical analysis was performed using SPSS software (Chicago, IL). The average tumor latency period is depicted in a Kaplan-Meier curve and was analyzed by a log rank test. The tumor and lesion incidences were analyzed by chi-square. Data were expressed as mean ± SEM. Data were considered statistically significant when p < 0.05.

## Results

### Incidence of palpable tumors

**Figure 1:**
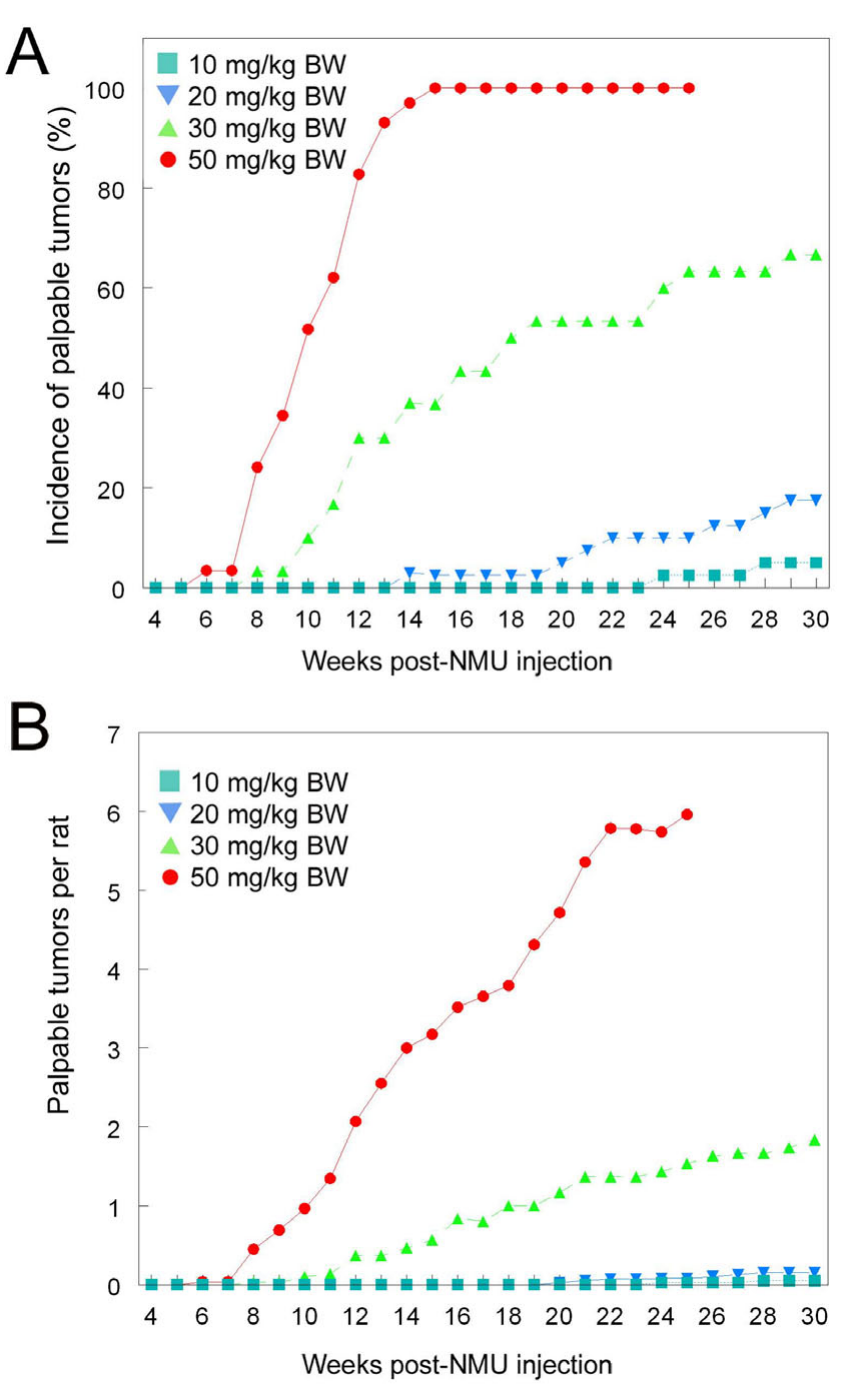
**Incidence of mammary gland tumors developing in Wistar-Furth female rats following different doses of NMU**. A: total tumor incidence; B: Average number of mammary gland tumors developing in female rats following different doses of NMU. n = 29–40/group.

Figure 1A shows that when the NMU dose was raised from 10 mg to 50 mg NMU/kg BW, the palpable tumor incidence increased accordingly, as did the average tumor number *per *rat (Figure 1B). When the dose was increased from 10 mg NMU/kg BW through 50 mg NMU/kg BW there was an approximate 6-fold increase in tumor number.

### Tumor latency

The first palpable tumors were evident at 24, 14, 8 and 6 weeks p.i. for the animals exposed to 10, 20, 30 and 50 mg NMU/kg BW, respectively. In addition, we calculated the average tumor latency for each group and found a decrease in tumor latency with increasing NMU dose. The average latency in weeks was 26.00 ± 2.00 for 10 mg NMU/kg BW, 22.57 ± 1.85 for 20 mg NMU/kg BW, 15.85 ± 1.32 for 30 mg NMU/kg BW and 10.48 ± 0.40 for 50 mg NMU/kg BW.

**Figure 2:**
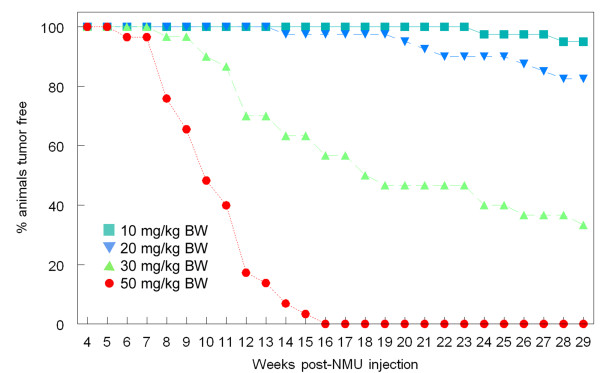
**Incidence of palpable tumors as a function of time after NMU injection**.

Figure 2 shows a Kaplan-Meier graph depicting the tumor latency period for all doses. A log rank analysis showed a significantly shorter latency in the group treated with 50 mg NMU/kg BW compared to all other groups (p < 0.0000). Additionally, latency was significantly shorter in the 30 mg NMU/kg BW group than in the 20 mg NMU/kg BW (p < 0.0000) and 10 mg NMU/kg BW (p < 0.0000) groups. There was no significant difference in tumor latency between the 10 mg NMU/kg BW and 20 mg NMU/kg BW dose.

### Histopathology of tumors

Histological evaluation of the tumors revealed that the two lower doses (10 mg NMU/kg BW and 20 mg NMU/kg BW) exclusively generated ductal carcinomas *in situ *(DCIS). Compared to tumors formed after exposure to 10 mg NMU/kg BW, tumors generated following exposure to 20 mg NMU/kg BW tended to show more features characteristic of an invasive phenotype such as subtle tumor growth projections into the normal tissue. Tumors from animals exposed to 30 mg NMU/kg BW were predominately DCIS with some showing invasive tendencies. In addition, one animal produced a DCIS with a fibroadenoma while another generated a fibroadenoma with an adenocarcinoma. Histology similar to the 30 mg NMU/kg BW group was seen in tumors excised from animals dosed with 50 mg NMU/kg BW.

### Aberrant structures in whole-mounted mammary glands

Microscopic inspection of the abdominal-inguinal whole-mounted mammary glands revealed a number of lesions and other epithelial abnormalities at all doses tested (Table 1). For the 12 and 22 weeks p.i. groups, the data are presented as incidence per gland as the left-side abdominal-inguinal mammary glands were surgically removed at 12 weeks after which the contralateral gland was removed at sacrifice (22 weeks). This paired data set showed that the aberrant structure incidence increased with NMU dose. In addition, their incidence increased over time with more aberrant structures at 22 weeks compared to 12 weeks for all doses except 10 mg NMU/kg BW. No lesions or other abnormal structures were identified in the mammary glands from rats treated with 10 mg NMU/kg BW at these early time points.

**Table 1:** Incidence of microscopic lesions and other microscopic abnormalities in NMU-treated whole mounted abdominal-inguinal mammary glands

NMU dose (mg/kg BW)	12 weeks incidence (%) ^Ω^	22 weeks incidence (%)^Ω^	25–30 weeks incidence (%) ^§^
0	0/6 (0)	0/5 (0)	0/9 (0) ^ab^

10	0/6 (0)	0/6 (0)	2/38 (5.3) ^a^

20	0/6 (0)	1/6 (16.6)	9/37 (24.3) ^bc^

30	2/5 (40.0)	3/5 (60.0)	9/27 (33.3) ^c^

50	4/5 (80.0)	3/4 (75.0)	21/26 (80.8) ^d^

Ω indicates that the incidence was calculated 'per mammary gland' whereas § denotes the incidence calculated using abdominal-inguinal mammary glands from the left and right side of each animal. Different superscripts denote statistically significant differences (p < 0.05).

Thus, at the end of the experiments (25 weeks p.i. for 50 mg NMU/kg BW, 30 weeks p.i. for all other groups) analysis of left and right side abdominal-inguinal mammary glands for each animal showed a similar NMU dose-dependant incidence increase (Table 1). The incidence of abnormalities in the 50 mg NMU/kg BW group was significantly higher than in all other groups (p < 0.005) while the incidence in the 10 mg NMU/kg BW group was statistically lower than in the 20 mg NMU/kg BW (p = 0.02) and 30 mg NMU/kg BW (p = 0.003) groups. Interestingly, this time point was the only one where we observed aberrant structures in the whole-mounted mammary glands of animals from the 10 mg NMU/kg BW group where the incidence reached 5.3%.

### Classification of aberrant microscopic structures

To better understand the events occurring in the mammary gland before palpable tumors became evident, we performed histological evaluations of the aberrant structures found in the whole-mounted mammary glands from all doses and time points. As the 10 mg and 20 mg NMU/kg BW groups gave the lowest incidence of aberrant structures in whole mounted mammary glands (5.3% and 24.3% respectively), we performed histological evaluations of all the aberrant structures found in these whole mounts. In addition, we evaluated all lesions and other abnormalities found at the two higher doses (30 mg and 50 mg NMU/kg BW) at 12 and 22 weeks p.i. Finally, we surveyed a sub-set of aberrant structures from the two higher NMU doses at the last sacrifice point (25 and 30 weeks, respectively) to determine whether the histological classification of these structures changed as the carcinogenic process progressed.

### 10 and 20 mg NMU/kg BW group

**Figure 3:**
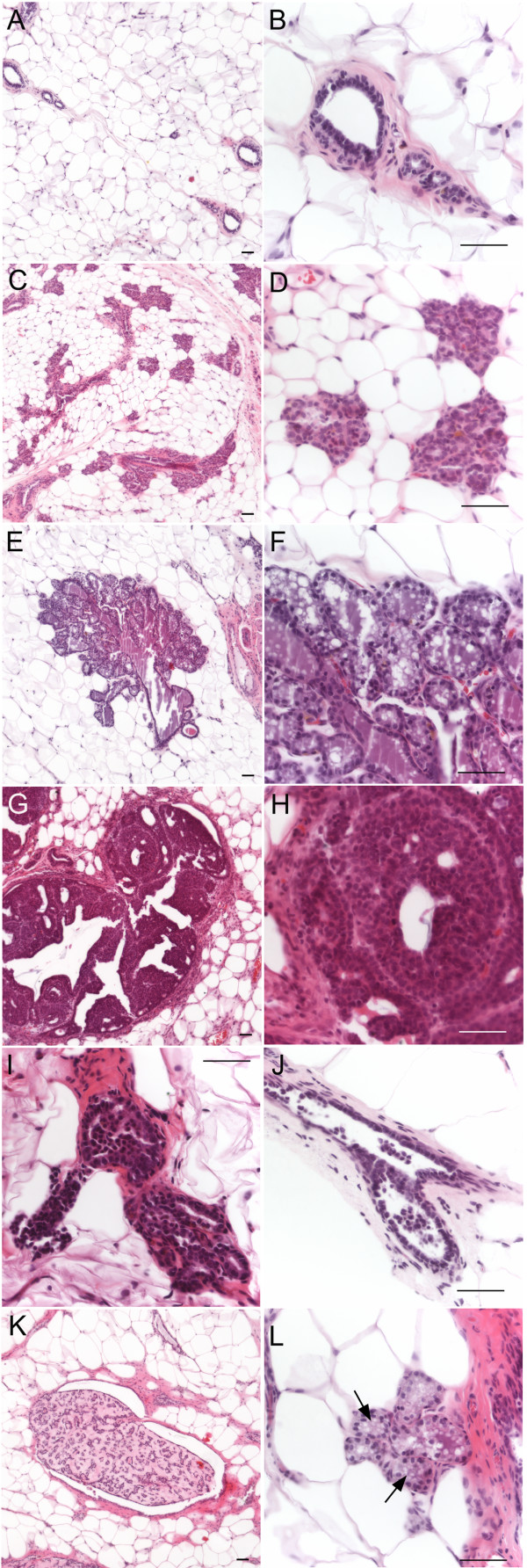
**H+E staining of mammary gland microscopic lesions and other microscopic abnormalities**. When compared to normal mammary gland (A, B), some of the glands show an increased number of acini which are normally only seen during pregnancy and lactation (C, D). Ducts that have further differentiated into lobular units with secretion are striking (E, F). Neoplasias include DCIS (G, H) and ductal hyperplasias (I). Sloughing of epithelial cells into the duct (J) is also observed. At higher doses of NMU (30 mg and 50 mg/kg BW), benign fibroadenomas and papillomas (K) and secretion into the duct (L) are observed. Scale bar: 50 μm

A number of morphological changes were observed in these glands as outlined in Table 2 and Figure 3. The most prevalent abnormality for both NMU doses was a change in the structure of the tissue that closely resembled a pregnant or lactating mammary gland phenotype (Figure 3C and 3D). That is, compared to a normal control mammary gland (Figure 3A and 3B), some mammary glands contained an increased number of acini (Figure 3E and 3F). In some cases, the epithelium had also further differentiated into a lobular unit composed of acini which were filled with secretion (Figure 3E and 3F). Closer examination revealed that the secretion, which appears pink in the H&E staining, contained fatty globules characteristic of milk production (Figure 3F).

**Table 2:** Classification of microscopic lesions and other microscopic abnormalities: low dose NMU-treated whole mounted mammary glands

	12 weeks				22 weeks				**25 and 30 weeks**^**§**^			
**Dose** **(mg/kg BW)**	**10**	**20**	**30**	**50**	**10**	**20**	**30**	**50**	**10**	**20**	**30**	**50**

Lactational change	0	0	0	0	0	0	1	2	2	4	1	0

Increased acini #	0	0	0	3	0	1	1	1	0	1	0	0

Hyperplasia	0	0	1	1	0	0	0	2	0	3	3	1

DCIS	0	0	0	1	0	1	1	0	0	2	7	18

^§^30 mg/kg BW animals were sacrificed at 30 weeks p.i. whereas 50 mg/kg BW animals were sacrificed at 25 weeks p.i. One abnormal structure per animal was selected for analysis at these time points compared to all structures being analyzed for all other groups.

**Figure 4:**
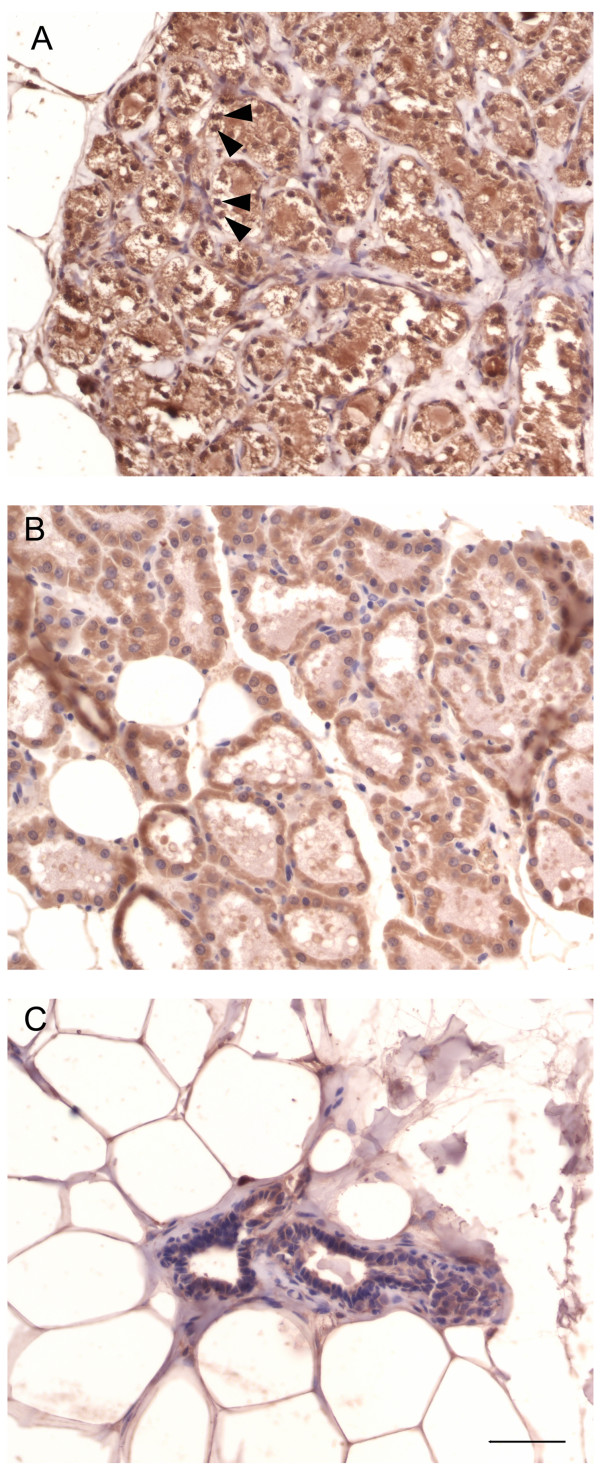
**Immunolocalization of α-lactalbumin in mammary glands**. In glands with the lactating phenotype (A), α-lactalbumin was localized to cells in the lobular units (arrowheads) and the secretion within the ducts. The positive control lactating mammary gland showed similar staining (B), while the normal virgin mammary gland was negative (C). Scale bar: 50 μm

These lactational changes in the virgin mammary glands were further characterized by performing immunostaining using an antibody raised against α-lactalbumin, a milk protein. In the glands exhibiting the lactating phenotype we found that the cells within the lobular units and the secretion within the ducts showed positive immunostaining (Figure 4A). A similar staining pattern was seen in a positive control lactating rat mammary gland (Figure 4B), confirming that the virgin glands had indeed altered their phenotype and were producing milk. Accordingly, a control virgin mammary gland did not stain with this antibody (Figure 4C) confirming that the antibody reacted specifically with the α-lactalbumin antigen.

Other morphological changes we observed were DCIS (Figure 3G and 3H) and hyperplastic ducts (Figure 3I); the latter were defined as ducts containing more than 3 layers of epithelial cells [14]. Finally, some ducts had an increase in the number of cells within the lumen of the epithelial ducts (Figure 3J). Immunostaining for pan-keratin revealed that this cell population included epithelial cells which had apparently sloughed into the lumen from the epithelial duct walls (data not shown). This phenomenon was not observed in control tissue.

**Figure 5:**
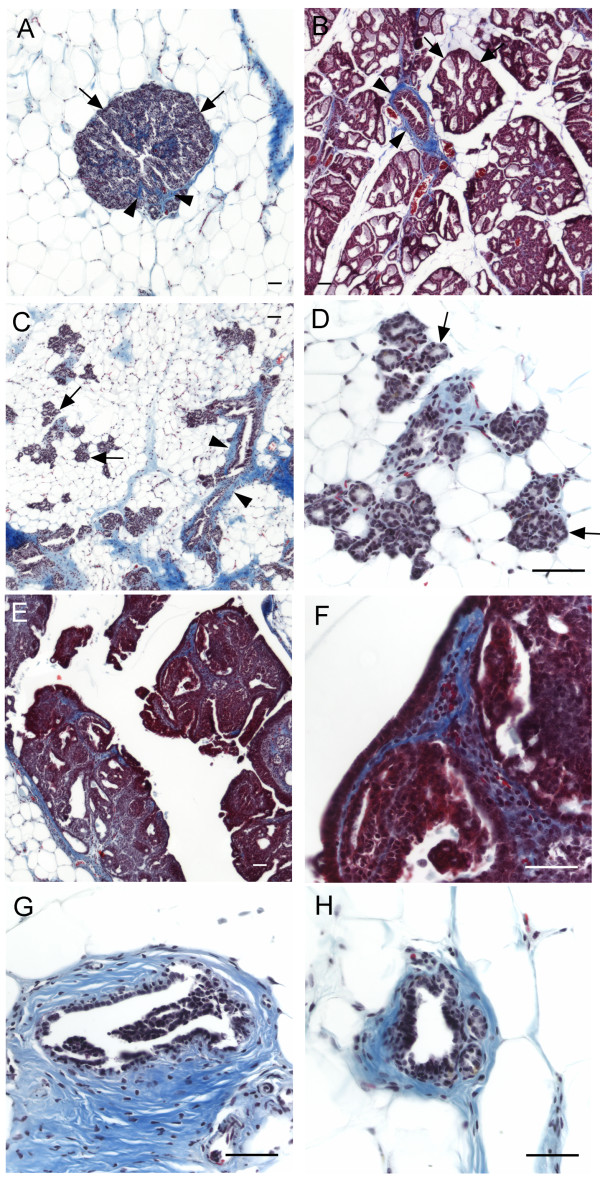
**Deposition of collagen (blue) in mammary glands of rats stained with Masson's Trichrome**. In glands with the lactating phenotype (A) and increased number of acini (C and D), collagen was found surrounding larger ducts (arrowheads) and not associated with acinar structures (arrows); this pattern was also evident in positive control lactating mammary gland (B). Collagen surrounded DCIS structures (E) and was found within the carcinoma (F). Ducts containing sloughed epithelial cells showed more collagen deposition surrounding the duct (G) than that seen in normal tissue (H). Scale bar: 50 μm

In order to determine whether the observed structural epithelial changes were accompanied by changes in the mammary gland stroma we stained for collagen content in our samples using Masson's Trichrome method. There was little collagen deposition around the acini of the glands exhibiting the lactating phenotype (Figure 5A); this pattern resembled that found in the positive control lactating rat mammary gland (Figure 5B). In tissues showing an increased number of acini, collagen deposition was most pronounced around the ducts and was relatively low around the acinar structures (Figure 5C and 5D). Some collagen was deposited around the hyperplastic and DCIS structures (Figure 5E); the DCIS also showed an increased deposition of collagen within the tumor (Figure 5F). Ducts containing the sloughed epithelial cells in the lumen were surrounded by stroma containing more collagen (Figure 5G) when compared to ducts from normal glands (Figure 5H).

Interestingly, no abnormal structures were seen in the animals injected with 10 mg NMU/kg BW which had palpable tumors. A similar pattern was observed in rats injected with 20 mg NMU/kg BW with only one exception: a palpable tumor and lactational change structure were found in one rat of this group.

### 30 and 50 mg NMU/kg BW group

Neoplastic and non-neoplastic features were observed in the mammary glands of rats treated with 30 mg or 50 mg NMU/kg BW as early as 12 weeks p.i. (Table 2 and Figure 3). In the 50 mg NMU/kg BW group, DCIS and hyperplasias were observed at 12 weeks p.i. alongside other structural alterations, such as increased numbers of epithelial structures resembling acini. At 22 weeks p.i., hyperplastic ducts and acini persisted. In addition, we observed secretion in ducts with otherwise normal histology while several ducts were found to have undergone morphological changes making their appearance resemble those found in a lactating gland. These changes were similar to those observed at the two lower doses of NMU. At 25 weeks p.i. the majority of abnormalities within the mammary gland were diagnosed as DCIS. However, there were also a number of benign lesions (fibroadenoma and papilloma types, Figure 3K) and hyperplastic ducts. Luminal secretion (Figure 3L) and sloughing of epithelial cells into the duct were also present.

Secretion into the ducts was also observed in the mammary glands from animals treated with 30 mg NMU/kg BW at 12 and 22 weeks p.i.; however, morphological changes consistent with lactation were only noted at later time points (22 and 30 weeks p.i.). These changes were also accompanied by a transient increase in the number of acini within the mammary gland at 22 weeks p.i. At this dose, ductal hyperplasias were also observed although these were only apparent at the earliest and latest time points. Furthermore, DCIS were apparent at this dose at the later time points, while benign fibroadenoma lesions were found at 30 weeks p.i. only.

At these doses of NMU, Trichrome staining of tissue exhibiting a lactating phenotype, increased number of acini, hyperplasias or DCIS revealed similar results to those reported for the two lower NMU doses. In addition, in tissues with ducts showing secretion into the lumen, collagen deposition was most pronounced around the larger ducts.

For the 30 mg NMU/kg BW group, similar to the lower NMU doses, aberrant structures were usually observed in animals that did not develop palpable tumors and conversely, animals that developed palpable tumors did not show any other obvious epithelial abnormalities. There was only oneexception: a palpable tumor and ductal hyperplasia were found in the same animal. Palpable tumor incidence reached 100% in the 50 mg NMU/kg BW animals, thus all aberrant structures found in this group were coincident with tumor formation.

## Discussion

This study attempts to fill the need for an in depth histopathological analysis of the mammary gland in Wistar Furth rats after exposure to low doses of NMU. It has been reported that female rats from susceptible strains develop mammary tumors when exposed to 50 mg/kg BW of the commonly used carcinogen, NMU [1,9,16-18]. Herein, rats from the susceptible Wistar-Furth strain were exposed to NMU at lower, and presumably, less carcinogenic doses. Following exposure to low doses of NMU (10 mg and 20 mg/kg BW), Wistar-Furth rats developed far fewer mammary gland carcinomas than those observed in animals treated with higher doses of carcinogen (30 mg and 50 mg NMU/kg BW). Furthermore, the latency to the first palpable tumor for the low dose animals was substantially longer than in those receiving higher doses of NMU (14–24 weeks *vs *6–8 weeks, respectively). However, a number of microscopic abnormalities were apparent in whole mounted mammary glands collected from the low dose animals while histological analysis showed that these ranged from simple ductal hyperplasias and DCIS to non-neoplastic structural changes. In the animals exposed to 10 mg or 20 mg NMU/kg BW, two distinct types of histological changes were observed, namely, (i) an increase in the number of acinar structures often accompanied by secretion into the lumen which is normally associated with pregnancy and lactation, and (ii) an increase in the number of epithelial cells sloughing into the lumen of the epithelial ducts.

There were also notable changes in collagen deposition associated with these structures compared to those seen in the normal rat mammary gland. The presence of non-neoplastic changes and alterations in collagen deposition are remarkable as they represent changes in tissue architecture. This is consistent with those theories of carcinogenesis that stress the role of tissue interactions in neoplastic development [17-19].

Changes in mammary gland architecture have been noted with other treatments. For example, our previous studies revealed that rodents exposed perinatally to the xenoestrogen bisphenol A showed changes in mammary gland architecture in adulthood when they developed intraductal hyperplasias with epithelial cells present inside the ductal lumen [20]. Furthermore, these bisphenol A-exposed animals exhibited changes in collagen localization as early as embryonic day 18 compared to unexposed control animals [21]. Also, it was observed that certain biochemical alterations of the extracellular matrix (e.g. by the addition of laminin) affected the expression of milk proteins in the mammary gland [22].

A reduced-dose NMU model provides an ideal platform for evaluating the tumor susceptibility of the rat mammary gland, and provides a baseline that can be used to model and predict the response to an NMU challenge to animals which are predisposed to developing cancer due to environmental insults. Based on the empirical finding that 10 mg and 20 mg NMU/kg BW caused more than a 50% lower tumor incidence compared to 50 mg NMU/kg BW (Figure 1; Table 1), and a significant increase in latency, we consider both low doses as suitable for use in future studies designed to evaluate whether certain treatments increase the propensity to mammary cancer.

The present studies highlight the importance of considering other, more subtle, effects caused by carcinogen exposure, such as the formation of microscopic dysplastic and neoplastic lesions. It is important to note that both the microscopic analysis and the length of this study showed findings that could have been overlooked in shorter studies and in those using only palpation as the measured endpoint. For instance, out of 40 animals in the lowest dose of NMU only 2 exhibited palpable tumors, however 2 other animals in this group developed non-neoplastic lesions. We conclude that all doses used in this study showed some carcinogenic effect and therefore a true "non-carcinogenic" NMU dose could not be defined. Studies claiming to use such a dose should be interpreted with caution [23,24].

A dose-response study in Sprague-Dawley rats revealed that the number of tumors increased and the latency decreased when the dose was increased from 25 mg NMU/kg BW to 75 mg NMU/kg BW [7]. No differences in tumor incidence, number or latency were noticed between animals injected with 50 mg NMU/kg BW at 28, 35 or 42 days of age. This study reinforces the importance of considering the rat strain selected when interpreting data given that the Sprague-Dawley rats injected with 50 mg NMU/kg BW at 42 days of age showed a lower tumor incidence and tumor burden than the Wistar-Furth rats used in our study. Similarly, an additional dose-response study highlights the importance of the route of NMU exposure as the Sprague-Dawley rats receiving an intravenous infusion of NMU at 50 days of age seemed to develop tumors faster than those Wistar-Furth rats in this study that were injected intraperitoneally; nevertheless, the overall tumor burden appeared similar [9]. Finally, we wish to highlight that this work characterizes the response of an inbred rat strain offering the possibility of using heterotypic tissue recombinations to better understand the role of the various mammary gland cell populations during the carcinogenic process [15].

## Conclusion

The results presented herein indicate that when rats from a susceptible strain are injected with a low dose of the carcinogen NMU, such as 10 mg/kg BW, and they are monitored for up to 7 months after exposure, they are still capable of developing mammary tumors. Thus, we have now established a useful "baseline effect" in the mammary gland against which any treatment aimed at increasing or decreasing tumor susceptibility in Wistar-Furth rats can be compared. To our knowledge, this is the first in-depth histological study of low dose NMU effects on the rat mammary gland. Finally, considering differences in tumor incidence and latency, we wish to stress the importance of conducting dose-response studies in the animal strain of interest as susceptibility between rat strains varies significantly.

## Competing interests

The authors declare that they have no competing interests.

## Authors' contributions

TJM performed all procedures and analysis and drafted the manuscript. MVM participated in the study design, helped coordinate the experiments and helped draft the manuscript. AAU confirmed all histological findings. AMS and CS conceived of the study, and participated in its design and coordination and helped to draft the manuscript. All authors read and approved the final manuscript.

## Pre-publication history

The pre-publication history for this paper can be accessed here:

http://www.biomedcentral.com/1471-2407/9/267/prepub
